# Natural Protein Intake in Children with Phenylketonuria: Prescription vs. Actual Intakes

**DOI:** 10.3390/nu15234903

**Published:** 2023-11-23

**Authors:** Alex Pinto, Anne Daly, Júlio César Rocha, Catherine Ashmore, Sharon Evans, Fatma Ilgaz, Mary Hickson, Anita MacDonald

**Affiliations:** 1 Birmingham Women’s and Children’s Hospital, Birmingham B4 6NH, UK; a.daly3@nhs.net (A.D.); catherine.ashmore@nhs.net (C.A.); evanss21@me.com (S.E.); fatma.celik@hacettepe.edu.tr (F.I.); anita.macdonald@nhs.net (A.M.); 2 Plymouth Institute of Health and Care Research, Faculty of Health, University of Plymouth, Plymouth PL6 8BH, UK; mary.hickson@plymouth.ac.uk; 3 Nutrition and Metabolism, NOVA Medical School (NMS), Faculdade de Ciências Médicas (FCM), Universidade Nova de Lisboa, 1169-056 Lisboa, Portugal; rochajc@nms.unl.pt; 4 CINTESIS@RISE, Nutrition and Metabolism, NOVA Medical School (NMS), Faculdade de Ciências Médicas, (FCM), Universidade NOVA de Lisboa, 1169-056 Lisboa, Portugal; 5 Reference Centre of Inherited Metabolic Diseases, Centro Hospitalar Universitario de Lisboa Central, 1169-045 Lisboa, Portugal; 6 Department of Nutrition and Dietetics, Faculty of Health Sciences, Hacettepe University, 06100 Ankara, Turkey

**Keywords:** phenylketonuria, fruits, vegetables, metabolic control, phenylalanine

## Abstract

In phenylketonuria (PKU), an important component of the UK dietary management system is a 50 mg phenylalanine (Phe)/1 g protein exchange system used to allocate the Phe/natural protein intakes according to individual patient tolerance. Any foods containing protein ≤ 0.5 g/100 g or fruits/vegetables containing Phe ≤ 75 mg/100 g are allowed without measurement or limit. In children with PKU, we aimed to assess the difference between the prescribed natural protein intake and their actual consumed intake, and to calculate the natural protein/Phe intake from foods given without measurement or restriction. Over a 6-month duration, three one-day diet diaries were collected every month by caregivers of children with PKU at the beginning of a follow-up study. Dietary intakes of Phe, as well as natural and total protein intakes, were calculated using Nutritics^®^ (v5.09). Weekly blood Phe spots were collected by caregivers. The target blood Phe level was ≤360 μmol/L for ages up to 12 years and ≤600 μmol/L for ages ≥12 years. Sixteen early treated children (69% females) with PKU were recruited. The median age was 11 years (range: 9–13), and most had classical PKU (n = 14/16). A median of 18 (range 12–18) one-day diaries and 22 blood spots were analysed for each subject over 6 months. The median prescribed natural protein was 6 g/day (range: 3–27), but when calculated, the actual median intake from all foods consumed was 10 g/day (range: 4–37). The median prescribed Phe was 300 mg/day (range: 150–1350), but the actual median intake was 500 mg/day (range: 200–1850). The median difference between the prescribed and actual natural protein daily intakes was +4 g/day (range: −2.5 to +11.5), with a median percentage increase of 40% for natural protein/Phe intake (*p* < 0.001). The median blood Phe level was 250 μmol/L (range 20–750), with 91% of blood Phe levels within the target range. Only one patient (11 years) had less than 75% of their blood Phe levels within the target range. The UK Phe exchange system provides flexibility in the dietary management of PKU. With this method, the actual natural protein intake was 167% higher than the prescribed amount. Although this led to a variable daily protein intake, the majority of children (n = 15/16) experienced no deterioration in their metabolic control.

## 1. Introduction

Phenylketonuria (PKU; OMIM 261600) is a rare inherited metabolic disorder caused by a deficiency of phenylalanine (Phe) hydroxylase, an enzyme which catalyses the conversion of the essential amino acid Phe into tyrosine [1,2]. If left untreated, Phe increases in the blood and brain, leading to severe clinical manifestations, including irreversible brain damage, intellectual disability, microcephaly, seizures, autism, motor deficits, eczematous rash, and behavioural problems [2,3]. These are successfully prevented by a natural protein/Phe-restricted diet, commenced immediately following newborn screening in the first weeks of life [3]. 

Dietary management consists of: (1) limited and controlled amounts of natural protein sources primarily sourced from plant foods; (2) unrestricted amounts of special low-protein foods, fruits and vegetables containing Phe ≤ 75 mg/100 g; and (3) low/Phe-free protein substitutes that provide the majority of the nitrogen source. The level of natural protein restriction depends on individual tolerance [4], which is influenced by the patient’s phenotype, age, growth status, use of adjunct therapies, and dosage of protein equivalent from protein substitute [4,5]. Recently, a systematic review and meta-analysis of 37 studies showed that natural protein tolerance gradually increases with age: from ≈5 g/day in infancy to 18 g/day at the beginning of puberty, reaching its highest level at the end of adolescence (32 g/day) [4]. However, most children aged ≤ 12 years with classical PKU tolerate ≤ 10 g/day of natural protein (500 mg/day of Phe) [5]. Special low-protein foods (e.g., bread, pasta) provide extra energy and aid adherence by adding variety.

In the UK during the early 1990s, a Phe-restricted diet was recommended for life [6]. However, long-term adherence to dietary restrictions is challenging [7,8,9,10], with studies consistently showing a decline in dietary adherence rates as patients age [9,10], particularly from the start of adolescence [7,9,10]. Inability to adhere to dietary treatment causes higher blood Phe concentrations [11], leading to sub-optimal neurocognitive outcomes and poorer executive function and academic achievement [12,13,14,15,16,17,18]. 

The main reasons for low dietary adherence are attributed to the following factors: limited food choices, poor palatability of special low-protein foods and protein substitutes, onerous routines, impaired dietary socialization, lack of access to special low-protein foods, accidental aspartame consumption, and inadequate patient/caregiver nutrient literacy. Additionally, poor adherence is associated with a more relaxed dietary approach and increased dietary errors arising from unsatisfactory dietary knowledge. In contrast, healthcare professionals should ensure that dietary treatment is not ‘stricter than necessary’, with minimal dietary calculations or protein counting for patients and their caregivers [19,20,21].

In clinical practice, natural protein/Phe is allocated using different approaches. The UK dietary system assumes that the amount of food that provides 1 g of protein is equivalent to 50 mg Phe, using an ‘exchange’ system. An alternative method is to calculate Phe from all foods eaten in the diet. Every method has its own advantages and limitations, but there is little evidence to show that any one method is better than another [5,21].

Using this system, any foods containing Phe ≤ 25 mg/100 g are given without measurement. These foods include special low-protein foods, starches, sweets, and sugars [5]. In 1996, MacDonald et al. [22] were the first to demonstrate that an increase in natural protein of 50% from ‘exchange-free’ foods with a low-Phe content, including foods such as butter, some starches, and fruits and vegetables containing Phe ≤ 75 mg/100 g of food, did not have a significant impact on blood Phe control. In a second prospective study, the same authors investigated the impact of free use of fruits and vegetables containing four different amounts of Phe on blood Phe levels [23]. It was demonstrated that unlimited consumption of fruits and vegetables containing Phe 51–75 mg/100 g (except for potatoes) was safe, and children with PKU could maintain metabolic stability. This tolerance was attributed to the poor Phe bioavailability from fruits and vegetables due to their low protein digestibility. This was confirmed by a recent randomised controlled study in patients with PKU [24] that suggested a greater impact of animal protein compared to vegetable protein when they provided the same amount of Phe. Based on the findings of MacDonald et al., researchers from Germany and Switzerland also investigated the effects of ‘diet liberalization’ with unlimited fruit and vegetable (Phe ≤ 75 mg/100 g) intake [25,26] and those of a ‘simplified’ diet [27]. Despite a significant increase in total Phe intake (+58–70 mg/day) [25,26], no adverse impact on blood Phe control was observed in both the short and long term [25,26,27]. The 2017 European PKU guidelines recommended that fruits and vegetables containing Phe ≤ 75 mg/100 g (except for potatoes) could be given without measurement in a Phe-restricted diet [3]. This simplified dietary approach has also been accepted and implemented in Australia and the USA [19,20,21].

The source of nitrogen in the protein substitute prescribed may also increase Phe intake. Casein glycomacropeptide (CGMP), derived from whey protein, is considered to have improved palatability compared to Phe-free amino acids [28,29]. It was first used as a protein substitute in 2008 in the USA but was not introduced in the UK until 2017 [30]. It is low in aromatic amino acids but contains residual amounts of Phe and tyrosine, typically providing 1.8 mg Phe/1 g of protein equivalent [29,31]. Several studies found higher blood Phe levels when patients took CGMP [28,30,32,33], but only three randomised controlled studies [28,30,33] in children from the same patient cohort showed that this had a statistically significant impact on blood Phe levels. When the amount of Phe from CGMP was not reduced from the daily Phe allowance, blood Phe levels were 18% higher in children with PKU [28].

There is inadequate information about the amino acid content of low protein foods due to the high cost of amino acid analysis, limited protein/aspartame food-labelling requirements, or low relevance of amino acid/protein content of these foods for the general population. Consequently, incomplete information may lead to the miscalculation of natural protein intake. Previous studies have also shown a high rate (>50%) of protein- labelling errors (e.g., unclear, misleading, inaccurate, or incomplete information) affecting interpretation of protein content of packaged manufactured foods on supermarket websites [34]. Ninety per cent of patients with PKU or their caregivers reported that they experienced problems with food labelling e.g., difficulties with interpreting food protein exchanges from food labels or labels containing misleading or confusing information [35].

Overall, in PKU, the prescribed amount of natural protein/Phe may not reflect the actual dietary intake due to differences in nutrient calculation practices e.g., incorporation of Phe from fruits and vegetables, Phe content from special low-protein products, CGMP protein substitutes, inaccurate or incomplete protein information on food labels, unidentified aspartame in draft soft drinks, and patient adherence or motivation to follow dietary prescriptions accurately. However, determining any differences in calculating Phe (protein) intake is important. This may improve blood Phe control and enable health professionals to accurately estimate the maximum Phe tolerance of patients. 

The aims of this study were to assess, in patients with PKU, the difference between the prescribed natural protein intake and their actual consumed intake, and to calculate the natural protein/Phe intake during a 6-month period using foods from the UK system given without measurement or restriction. 

## 2. Materials and Methods

### 2.1. Study Participants

Patients were recruited from the study sample of a randomised controlled cross-over trial conducted at Birmingham Children’s Hospital PKU clinic to investigate the impact of fruit and vegetable protein vs. milk protein on metabolic control in children with PKU [24]. The inclusion criteria have been previously described. In total, 16 patients aged between 6 and 13 years participated in the study.

### 2.2. Study Design

Patients were studied for an additional 6 months after the randomised controlled trial was completed, and the results were published [24]. During the 6-month follow-up period, monthly 3 one-day food diaries, including a detailed description of intake (e.g., food type, portion sizes), were obtained from all patients/caregivers to calculate the actual natural protein and Phe intakes. No further interventions were performed during the study period. Routine blood Phe samples were taken weekly.

All patients were prescribed natural protein according to their ‘assumed’ tolerance; defined as the maximum Phe intake maintaining blood Phe levels below the upper target limit. Special low-protein foods, foods containing protein <0.5 g/100 g, and fruit and vegetables containing Phe <100 mg/100 g were allowed without measurement as part of the intervention study. Natural protein was allocated in the diet using the UK 50 mg Phe exchange system, whereby 1 exchange is equivalent to 1 g of protein/50 mg of Phe. Patients were prescribed low-Phe/Phe-free protein substitutes during the study. The blood Phe levels recommended by the European PKU Guidelines (aged 0–11 years: ≤360 μmol/L, and aged ≥12 years: ≤600 μmol/L) [3] were used as the target therapeutic levels to define good metabolic control, according to routine clinical practice. 

### 2.3. Data Collection and Analysis

Data were collected between 29 January 2019 and 30 July 2021. An electronic software package (Nutritics^®^, v5.09, Dublin, Ireland) was used to analyse the monthly 3 one-day food diaries by AP. Natural protein and Phe intakes from all foods ingested were calculated, including foods permitted without measurement. Data on prescribed natural protein and Phe intakes were collected from the patient records. 

Patients provided weekly blood spots for Phe and tyrosine (Tyr) analyses, as per routine care. At least two fasting morning blood spots were collected on each filter card (Perkin Elmer 226, UK Standard NBS). Blood Phe levels were analysed using a 3.2 mm punch and MS/MS tandem mass spectrometry.

### 2.4. Statistical Analysis

Sample size calculations were performed for a previous randomised controlled trial, with a more detailed explanation provided in Pinto et al. [24]. Previous data were used to calculate the sample size, and a 40 µmol/L standard deviation in a patient’s blood Phe level was considered as a clinically relevant difference. In total, 13 subjects were required to obtain a statistical power of 90%. However, 16 subjects were recruited to allow for attrition. In this study, the descriptive analysis of continuous data is presented as median (range) or mean (SD), while the categorical variables are expressed as percentages. SPSS version 23.0 (IBM Company, Chicago, IL, USA) was used for the analysis. Normal distribution was tested using a Shapiro–Wilk test. Wilcoxon tests were performed to identify differences when non-normal distributions were found. The level of statistical significance was considered at *p* < 0.05.

### 2.5. Ethical Aspects

This project was approved on the 26 April 2019 by the East Midlands, Leicester South Research Ethics Committee with the reference number 19/EM/0073 and the Integrated Research Application System (IRAS) number 252561. It was also registered on clinicaltrials.gov, accessed on 25 October 2023 (No: NCT05249218). Informed consent was obtained from parents/caregivers, and age-appropriate assent was obtained from the patients. This study was performed according to Good Clinical Practice guidelines and the ‘Declaration of Helsinki’ (52nd WMA General Assembly, Edinburgh, UK, October 2000).

## 3. Results

### 3.1. Study Participants

Sixteen early and continuously treated patients with PKU, identified via newborn screening, participated in this study (11 females and five males). At baseline, the median age was 11 years (range: 6–13 years). Two patients were of Asian origin and the remaining were of European/Caucasian origin. Most patients had classical PKU, except for n = 2 patients who had mild PKU. Phenotype classification was defined according to genetic mutations or dietary Phe tolerance. The main characteristics of the study subjects are summarized in Table 1.

### 3.2. Natural Protein and Phe: Prescribed vs. Actual Intakes

A median of 18 (range: 12–18) daily diaries were analysed for each subject. Most patients completed three one-day diet diaries each month, but two patients only completed two daily diaries monthly throughout the study. The prescribed and actual natural protein/Phe intakes during the 6-month period are presented in Table 2. The median prescribed natural protein was 6 g/day (range 3–27 g/day), and the Phe intake was 300 mg/day (range: 150–1350 mg/day). The actual median intake of natural protein was 10 g/day (range: 4–37 g/day), and the Phe intake was 500 mg/day (range: 200–1850 mg/day). 

The median difference between the daily prescribed and actual intake of natural protein was calculated (Table 3). The actual natural protein intake was +4 g/day (range: −2.5 g to 11.5 g), and the Phe intake was +200 mg/day (range: −125 mg to 575 mg) higher than the prescribed amounts, respectively, corresponding to a median of 40% (range: −50% to 75%) difference between the two dietary calculations (*p* < 0.001). 

The overall intake of fruit and vegetables containing Phe <100 mg/100 g contributed a median of 2 g/day (range: 0–8) of natural protein. The remaining 2 g/day were sourced from special low-protein foods containing protein < 0.5 g/100 g or from additional protein eaten because of a natural protein/Phe calculation error. 

All natural protein intakes and prescriptions for all participants during the study are presented in Supplementary Table S1.

### 3.3. Metabolic Control

The blood Phe control of the study participants is described in Table 4. In total, subjects had a median of 22 blood spots during the 6-month period, which was equivalent to a weekly blood spot for each patient. The median blood Phe level was 250 μmol/L (range: 20–750 μmol/L). Patients had a median of 91% of blood Phe levels within target range, according to the recommendations of the European PKU Guidelines [3]. During the study period, patient 14 became less adherent to dietary treatment, with unsatisfactory blood Phe control (% of blood Phe levels within target = 59%). The median difference between the actual intake and prescribed amount (% increase) of protein for patient 14 was +3 g (33%), with fruit and vegetables containing Phe <100 mg/100 g contributing a median of 1.6 g/day (range: 0–2.3) of natural protein.

## 4. Discussion

In this study, the natural protein and Phe intakes of children with PKU were calculated from monthly three one-day food diaries during a 6-month period. These values were then compared with prescribed amounts of natural protein to investigate any differences that may affect blood Phe control. Our results showed that patients consumed 40% more natural protein (equivalent to an additional 4 g/day natural protein) than prescribed. Extra natural protein intake from fruit and vegetables containing Phe <100 mg/100 g added a median of 2 g/day. The remaining 2 g/day were sourced from unmeasured natural protein/Phe in special low-protein foods, foods containing <0.5 g of protein/100 g, or miscalculations of natural protein/Phe intake. Despite the statistically significant amount of additional Phe intake, metabolic control was maintained within the target recommendations for n = 15/16 (94%) patients, with a median of 91% of the blood Phe levels within the therapeutic target range during the study period. 

Since the first introduction of a Phe-restricted diet in the 1950s [36], the ultimate goal of management has been to achieve and maintain blood Phe levels within a safe target range. This strategy has been successful, transforming patient outcomes to achieve near normal neurocognition [3]. However, dietary treatment is associated with a substantial burden [37] although it remains the core treatment for the majority of patients [3,38]. Several adjunct or alternative therapies have been developed, such as sapropterin (BH4) or the enzyme substitution therapy ‘pegvaliase’ [2]. Unlike the Phe-restrictive diet, pharmaceutical treatments aim to improve Phe tolerance and consequently decrease the dietary burden for patients with PKU and their caregivers, but these treatments are not without disadvantages. Sapropterin treatment is only effective in responsive individuals, usually patients with milder PKU (around 30% of patients), and most still require some dietary Phe restrictions [38]. Enzyme substitution therapy is an invasive treatment that usually requires daily injections and is only licenced in Europe for patients aged 16 years and older with blood Phe levels ≥ 600 µmol/L. In addition, adverse events are commonly reported, ranging in severity (e.g., skin reactions, arthralgia, and anaphylactic responses). Many countries have limited access to its use [39,40,41,42]. 

The work of MacDonald and colleagues [22], along with subsequent studies, suggested that calculating Phe from all foods may ensure a more accurate estimation of Phe intake but can compromise dietary adherence due to unnecessary restrictions and extra burden. Our study confirmed that higher Phe intake compared to prescription did not impact on long-term metabolic control. This has particular importance for adolescents and adults who struggle with dietary treatment. A Phe-restricted diet is a burden for patients, their caregivers, and health professionals. Even in the UK, where an exchange system is used, the family of a child with PKU may spend up to 19 h/week on PKU-related care activities, but only two hours/week are spent on calculating and weighing food intake [43]. The time spent on dietary care is likely to be higher if the patients or caregivers must measure all Phe intake, negatively impacting the quality of life. Giving more ‘free’ food choices may help ease the burden of diet therapy. This effect was observed in patients with mild-PKU undergoing sapropterin treatment, following diet relaxation [7]. 

Previously, we observed that animal protein impacts blood Phe levels differently compared to vegetable sources [24]. The lack of impact of additional natural protein intake from fruits and vegetables on blood Phe levels has been associated with the low digestibility and absorption of protein from vegetable food sources [44]. The bioavailability of natural protein may be influenced by food processing, eating, and oral mastication [45,46]. Vegetable protein absorption may also be reduced by the presence of fibre that must be digested and by the hydrophobic-sheet structure of plant protein that facilitates aggregation, slowing protein utilisation. Anti-nutritional factors present in vegetables, such as phytic acid, protease inhibitors, hemagglutinins, glucosinolates, tannins, and gossypol may also cause endogenous losses, consequently decreasing protein absorption [47]. The impact may differ depending on individual tolerance, particularly for patients who have classical PKU. Microbiota may also play an important role in Phe digestion; consumption of plant protein has demonstrated a higher abundance of bacteroidetes and lower firmicutes [48].

Few studies have compared the actual protein or Phe intake versus the prescribed amount when using a Phe exchange system. Our results support previously published data [22], reporting that actual intakes could be ≈50% higher than prescribed when using a Phe exchange system where natural protein intake from fruits, vegetables, foods containing Phe ≤ 25 mg/100 g, and special low-protein foods is not calculated in the daily natural protein/Phe allowance. In this study, we demonstrated an increase of 40% in natural protein/Phe intake compared to patient prescriptions, ranging from −50% to 75%. Frequent (weekly) blood Phe monitoring showed no overall impairment in the quality of blood Phe control. These results are also in line with the findings of a large European survey conducted in 2011 that recruited approximately 2000 diet-treated patients with PKU from 10 centres [49]. The survey indicated that metabolic control in patients with PKU in Europe was similar irrespective of the method used to calculate dietary Phe intake (calculating all Phe intake vs. using a Phe exchange system). Our findings, together with evidence from previous studies, confirm that the use of a 50 mg Phe exchange system with more dietary choices from unrestricted foods is safe in PKU. Any higher blood Phe levels observed were associated with patients mistakenly or intentionally consuming additional Phe from higher protein foods or failing to take the prescribed amounts of protein substitute. Only one older patient failed to achieve satisfactory blood Phe control with only 59% of levels within target range. However, comparing their actual and prescribed protein intake showed similar differences to the overall group.

There are no reliable life-time data on the natural protein tolerance of patients with PKU, and there may be inter- as well as intra-variability. In a recent systematic review [4], there were only a few studies that reported both prescribed and actual intakes. There was also limited information about the natural protein intake consumed throughout life, particularly in adult patients with PKU. For optimal patient outcomes, including good metabolic control and adequate child and adolescent growth, as well as to study the effectiveness of pharmaceutical adjunct therapies, it is essential to define natural protein requirements by systematically assessing dietary intakes. 

### Limitations

There were limitations to this study. Completing three one-day diet diaries each month was time-consuming and is likely to have affected the accuracy of the quantities and number of foods that were reported. However, these diaries were always checked directly with the parents/caregivers by the researchers. They also record a specific time period and may not capture long-term food patterns like a food frequency questionnaire. Patients/caregivers may under report natural protein intake when using diet diaries to avoid disclosing to health professionals any protein-containing foods eaten in excess. Patients also completed their diet diaries over a 6-month period, which encompassed different seasons of the year, affecting food choice and availability, and potentially impacting daily protein intake. The study sample size was small, and the age range was narrow (6–13 years), which may have affected food choice and the quantity of food eaten.

## 5. Conclusions

The UK natural protein/Phe exchange system provides flexibility in the dietary management of PKU. With this system, the actual natural protein intake is almost double the prescribed amount. Although the system leads to a variable and higher-than-prescribed daily protein intake, no deterioration of metabolic control is observed. More data is needed about natural protein intake in patients with PKU in different stages of life in order to provide more information about protein tolerance throughout adolescence and adulthood.

## Figures and Tables

**Table 1:** Characteristics of patients with PKU participating in this study.

#	Age at Baseline (Years)	Gender	Mutations	Phenotype ^a^	N of Prescribed 1 g Protein Exchanges/Day ^b^
1	10	F	Unknown	cPKU	5
2	8	M	C1315+1G>A.p.? c.782G>A p (Arg261Gln)	cPKU	6.5
3	12	M	c.960G>C p. (Lys320Asn) c.728G>A (Arg243Gln)	mPKU	27
4	13	F	c.194T>C p. (Ile65Thr) C.1066-11G>Ap.?	mPKU	14
5	9	F	C194t>c c.1222C>T p.(Arg408Trp)	cPKU	6
6	12	M	c. 1066-11 G>A p. ? c. 912+1 G>A p. ?	cPKU	7.5
7	13	F	c.47_48del p. (Ser16*) c.1222C>T p.(Arg408Trp)	cPKU	4.5
8	9	M	c.1222C>T p. (Arg408Trp) c.1222C>T p. (Arg408Trp)	cPKU	3
9	6	F	c.745C>T p. (Leu249Phe) c.1315+1G>A.p.?	cPKU	5.5
10	12	F	c.558_559del p. (Trp187Glyfs*12) c.558_559del p. (Trp187Glyfs*12)	cPKU	4
11	12	F	c.782G>A p. (Arg261Gln) c.896T>G p. (Phe299Cys)	cPKU	7
12	12	F	c.558_559del p. (Trp187Glyfs*12) c.558_559del p. (Trp187Glyfs*12)	cPKU	4
13	11	F	c.782G>A p. (Arg261Gln) c.896T>G p. (Phe299Cys)	cPKU	6.5
14	11	F	Unknown	cPKU	6
15	9	M	c.926C>T p. (Ala309Val) c.1103A>G p. (Glu368Gly)	cPKU	4
16	10	F	c.782G>A p. (Arg261Gln) c.1222C>T p. (Arg408Trp)	cPKU	6

Abbreviations: PKU, phenylketonuria; F, female; M, male; cPKU, classical phenylketonuria; mPKU, mild phenylketonuria; N, number. ^a^ Patients’ phenotypes were defined according to genetic mutations, or based on their natural protein tolerance when mutations were not known. ^b^ One exchange is equivalent to 1 g of protein or 50 mg of Phe.

**Table 2:** Prescribed and actual intakes of natural protein and Phe during the study.

	Prescribed; Median (Range)		Actual Intake; Median (Range)	
	Natural Protein (g/day)	Phenylalanine (mg/day)	Natural Protein (g/day)	Phenylalanine (mg/day)
Month 1	6 (3–27)	300 (150–1350)	10 (5–31)	500 (250–1550)
Month 2			9 (4–35)	450 (200–1750)
Month 3			10 (5–33)	500 (250–1650)
Month 4			10 (4–34)	500 (200–1700)
Month 5			9.5 (4–31)	475 (200–1550)
Month 6			9 (4–37)	450 (200–1850)
Median (range)			10 (4–37)	500 (200–1850)

**Table 3:** Differences between total natural protein and Phe intakes and prescriptions.

	Mean (SD)	Median (Range)
Prescribed natural protein (g/day)	7 (6)	6 (3–27)
Actual natural protein intake (g/day)	11 (6)	10 (4–37)
Difference between prescribed and actual intakes of natural protein	4 (2.4)	4 (−2.5–11.5)
Prescribed Phe (mg/day)	367 (289)	300 (150–1350)
Actual Phe intake (mg/day)	563 (323)	500 (200–1850)
Difference between prescribed and actual intakes of Phe	197 (118)	200 (−125–575)

Abbreviations: Phe, phenylalanine; SD, standard deviation.

**Table 4:** Metabolic control of all participants during the study.

	Metabolic Control Parameters
Median (range) of blood Phe levels (μmol/L)	250 (20–750)
Mean (SD) of blood Phe levels (μmol/L)	271 (142)
% of blood Phe levels within target (0–11 years: <360 μmol/L; ≥12 years: <600 μmol/L)	91 (59–100)
Median (range) of blood Tyr levels (μmol/L)	50 (20–210)
Total number of blood spots for all subjects	348

Abbreviations: Phe, phenylalanine; Tyr, tyrosine; SD, standard deviation.

## Data Availability

Data are contained within the article and Supplementary Materials.

## Supplementary Materials

The following supporting information can be downloaded at: https://www.mdpi.com/article/10.3390/nu15234903/s1, Table S1: Natural protein and phenylalanine of all patients during the study—intake and prescribed.

## References

[1] Blau N., van Spronsen F.J., Levy H.L. (2010). Phenylketonuria. Lancet.

[2] van Spronsen F.J., Blau N., Harding C., Burlina A., Longo N., Bosch A.M. (2021). Phenylketonuria. Nat. Rev. Dis. Primers.

[3] van Wegberg A.M.J., MacDonald A., Ahring K., Bélanger-Quintana A., Blau N., Bosch A.M., Burlina A., Campistol J., Feillet F., Giżewska M. (2017). The complete European guidelines on phenylketonuria: Diagnosis and treatment. Orphanet J. Rare Dis..

[4] Pinto A., Ilgaz F., Evans S., van Dam E., Rocha J.C., Karabulut E., Hickson M., Daly A., MacDonald A. (2023). Phenylalanine Tolerance over Time in Phenylketonuria: A Systematic Review and Meta-Analysis. Nutrients.

[5] MacDonald A., van Wegberg A.M.J., Ahring K., Beblo S., Bélanger-Quintana A., Burlina A., Campistol J., Coşkun T., Feillet F., Giżewska M. (2020). PKU dietary handbook to accompany PKU guidelines. Orphanet J. Rare Dis..

[6] Phenylketonuria M.W.P. (1993). Recommendations on the dietary management of phenylketonuria. Arch. Dis. Child..

[7] Cazzorla C., Bensi G., Biasucci G., Leuzzi V., Manti F., Musumeci A., Papadia F., Stoppioni V., Tummolo A., Vendemiale M. (2018). Living with phenylketonuria in adulthood: The PKU ATTITUDE study. Mol. Genet. Metab. Rep..

[8] Cotugno G., Nicolò R., Cappelletti S., Goffredo B.M., Dionisi Vici C., Di Ciommo V. (2011). Adherence to diet and quality of life in patients with phenylketonuria. Acta Paediatr..

[9] García M.I., Araya G., Coo S., Waisbren S.E., de la Parra A. (2017). Treatment adherence during childhood in individuals with phenylketonuria: Early signs of treatment discontinuation. Mol. Genet. Metab. Rep..

[10] Jurecki E.R., Cederbaum S., Kopesky J., Perry K., Rohr F., Sanchez-Valle A., Viau K.S., Sheinin M.Y., Cohen-Pfeffer J.L. (2017). Adherence to clinic recommendations among patients with phenylketonuria in the United States. Mol. Genet. Metab..

[11] Walter J.H., White F.J. (2004). Blood phenylalanine control in adolescents with phenylketonuria. Int. J. Adolesc. Med. Health.

[12] Azen C.G., Koch R., Friedman E.G., Berlow S., Coldwell J., Krause W., Matalon R., McCabe E., O’Flynn M., Peterson R. (1991). Intellectual development in 12-year-old children treated for phenylketonuria. Am. J. Dis. Child..

[13] Bilder D.A., Noel J.K., Baker E.R., Irish W., Chen Y., Merilainen M.J., Prasad S., Winslow B.J. (2016). Systematic Review and Meta-Analysis of Neuropsychiatric Symptoms and Executive Functioning in Adults With Phenylketonuria. Dev. Neuropsychol..

[14] Chang P.N., Gray R.M., O’Brien L.L. (2000). Patterns of academic achievement among patients treated early with phenylketonuria. Eur. J. Pediatr..

[15] Hood A., Antenor-Dorsey J.A., Rutlin J., Hershey T., Shimony J.S., McKinstry R.C., Grange D.K., Christ S.E., Steiner R., White D.A. (2015). Prolonged exposure to high and variable phenylalanine levels over the lifetime predicts brain white matter integrity in children with phenylketonuria. Mol. Genet. Metab..

[16] Nardecchia F., Manti F., Chiarotti F., Carducci C., Carducci C., Leuzzi V. (2015). Neurocognitive and neuroimaging outcome of early treated young adult PKU patients: A longitudinal study. Mol. Genet. Metab..

[17] Thomas L., Olson A., Romani C. (2023). The impact of metabolic control on cognition, neurophysiology, and well-being in PKU: A systematic review and meta-analysis of the within-participant literature. Mol. Genet. Metab..

[18] Waisbren S.E., Noel K., Fahrbach K., Cella C., Frame D., Dorenbaum A., Levy H. (2007). Phenylalanine blood levels and clinical outcomes in phenylketonuria: A systematic literature review and meta-analysis. Mol. Genet. Metab..

[19] Bernstein L., Burns C., Sailer-Hammons M., Kurtz A., Rohr F. (2017). Multiclinic Observations on the Simplified Diet in PKU. J. Nutr. Metab..

[20] Hansen J., Hollander S., Drilias N., Van Calcar S., Rohr F., Bernstein L. (2020). Simplified Diet for nutrition management of phenylketonuria: A survey of U.S. metabolic dietitians. JIMD Rep..

[21] Sweeney A.L., Roberts R.M., Fletcher J.M. (2012). Dietary protein counting as an alternative way of maintaining metabolic control in phenylketonuria. JIMD Rep..

[22] MacDonald A., Rylance G., Hall S.K., Asplin D., Booth I.W. (1996). Factors affecting the variation in plasma phenylalanine in patients with phenylketonuria on diet. Arch. Dis. Child..

[23] MacDonald A., Rylance G., Davies P., Asplin D., Hall S.K., Booth I.W. (2003). Free use of fruits and vegetables in phenylketonuria. J. Inherit. Metab. Dis..

[24] Pinto A., Daly A., Rocha J.C., Ashmore C., Evans S., Jackson R., Payne A., Hickson M., MacDonald A. (2022). Impact of Fruit and Vegetable Protein vs. Milk Protein on Metabolic Control of Children with Phenylketonuria: A Randomized Crossover Controlled Trial. Nutrients.

[25] Rohde C., Mütze U., Schulz S., Thiele A.G., Ceglarek U., Thiery J., Mueller A.S., Kiess W., Beblo S. (2014). Unrestricted fruits and vegetables in the PKU diet: A 1-year follow-up. Eur. J. Clin. Nutr..

[26] Rohde C., Mütze U., Weigel J.F., Ceglarek U., Thiery J., Kiess W., Beblo S. (2012). Unrestricted consumption of fruits and vegetables in phenylketonuria: No major impact on metabolic control. Eur. J. Clin. Nutr..

[27] Zimmermann M., Jacobs P., Fingerhut R., Torresani T., Thöny B., Blau N., Baumgartner M.R., Rohrbach M. (2012). Positive effect of a simplified diet on blood phenylalanine control in different phenylketonuria variants, characterized by newborn BH4 loading test and PAH analysis. Mol. Genet. Metab..

[28] Daly A., Evans S., Chahal S., Santra S., Pinto A., Gingell C., Rocha J.C., van Spronsen F., Jackson R., MacDonald A. (2019). The Effect of Glycomacropeptide versus Amino Acids on Phenylalanine and Tyrosine Variability over 24 Hours in Children with PKU: A Randomized Controlled Trial. Nutrients.

[29] Ney D.M., Gleason S.T., van Calcar S.C., MacLeod E.L., Nelson K.L., Etzel M.R., Rice G.M., Wolff J.A. (2009). Nutritional management of PKU with glycomacropeptide from cheese whey. J. Inherit. Metab. Dis..

[30] Daly A., Evans S., Chahal S., Santra S., Pinto A., Jackson R., Gingell C., Rocha J., Van Spronsen F.J., MacDonald A. (2019). Glycomacropeptide: Long-term use and impact on blood phenylalanine, growth and nutritional status in children with PKU. Orphanet J. Rare Dis..

[31] Nakano T., Silva-Hernandez E.R., Ikawa N., Ozimek L. (2002). Purification of kappa-casien glycomacropeptide from sweet whey with undetectable level of phenylalanine. Biotechnol. Prog..

[32] Ahring K.K., Lund A.M., Jensen E., Jensen T.G., Brøndum-Nielsen K., Pedersen M., Bardow A., Holst J.J., Rehfeld J.F., Møller L.B. (2018). Comparison of Glycomacropeptide with Phenylalanine Free-Synthetic Amino Acids in Test Meals to PKU Patients: No Significant Differences in Biomarkers, Including Plasma Phe Levels. J. Nutr. Metab..

[33] Daly A., Evans S., Chahal S., Santra S., MacDonald A. (2017). Glycomacropeptide in children with phenylketonuria: Does its phenylalanine content affect blood phenylalanine control?. J. Hum. Nutr. Diet..

[34] Kraleva D., Evans S., Pinto A., Daly A., Ashmore C., Pointon-Bell K., Rocha J.C., MacDonald A. (2020). Protein Labelling Accuracy for UK Patients with PKU Following a Low Protein Diet. Nutrients.

[35] Hall I., Pinto A., Evans S., Daly A., Ashmore C., Ford S., Buckley S., MacDonald A. (2022). The Challenges and Dilemmas of Interpreting Protein Labelling of Prepackaged Foods Encountered by the PKU Community. Nutrients.

[36] Bickel H., Gerrard J., Hickmans E.M. (1953). Influence of phenylalanine intake on phenylketonuria. Lancet.

[37] Eijgelshoven I., Demirdas S., Smith T.A., van Loon J.M., Latour S., Bosch A.M. (2013). The time consuming nature of phenylketonuria: A cross-sectional study investigating time burden and costs of phenylketonuria in the Netherlands. Mol. Genet. Metab..

[38] Vockley J., Andersson H.C., Antshel K.M., Braverman N.E., Burton B.K., Frazier D.M., Mitchell J., Smith W.E., Thompson B.H., Berry S.A. (2014). Phenylalanine hydroxylase deficiency: Diagnosis and management guideline. Genet. Med..

[39] Gupta S., Lau K., Harding C.O., Shepherd G., Boyer R., Atkinson J.P., Knight V., Olbertz J., Larimore K., Gu Z. (2018). Association of immune response with efficacy and safety outcomes in adults with phenylketonuria administered pegvaliase in phase 3 clinical trials. eBioMedicine.

[40] Harding C.O., Amato R.S., Stuy M., Longo N., Burton B.K., Posner J., Weng H.H., Merilainen M., Gu Z., Jiang J. (2018). Pegvaliase for the treatment of phenylketonuria: A pivotal, double-blind randomized discontinuation Phase 3 clinical trial. Mol. Genet. Metab..

[41] Longo N., Zori R., Wasserstein M.P., Vockley J., Burton B.K., Decker C., Li M., Lau K., Jiang J., Larimore K. (2018). Long-term safety and efficacy of pegvaliase for the treatment of phenylketonuria in adults: Combined phase 2 outcomes through PAL-003 extension study. Orphanet J. Rare Dis..

[42] Thomas J., Levy H., Amato S., Vockley J., Zori R., Dimmock D., Harding C.O., Bilder D.A., Weng H.H., Olbertz J. (2018). Pegvaliase for the treatment of phenylketonuria: Results of a long-term phase 3 clinical trial program (PRISM). Mol. Genet. Metab..

[43] MacDonald A., Smith T.A., de Silva S., Alam V., van Loon J.M. (2016). The personal burden for caregivers of children with phenylketonuria: A cross-sectional study investigating time burden and costs in the UK. Mol. Genet. Metab. Rep..

[44] Suárez López M.M., Kizlansky A., López L.B. (2006). Assessment of protein quality in foods by calculating the amino acids score corrected by digestibility. Nutr. Hosp..

[45] Amigo L., Hernández-Ledesma B. (2020). Current Evidence on the Bioavailability of Food Bioactive Peptides. Molecules.

[46] Rein M.J., Renouf M., Cruz-Hernandez C., Actis-Goretta L., Thakkar S.K., da Silva Pinto M. (2013). Bioavailability of bioactive food compounds: A challenging journey to bioefficacy. Br. J. Clin. Pharmacol..

[47] Samtiya M., Aluko R.E., Dhewa T. (2020). Plant food anti-nutritional factors and their reduction strategies: An overview. Food Prod. Process. Nutr..

[48] Verduci E., Carbone M.T., Borghi E., Ottaviano E., Burlina A., Biasucci G. (2020). Nutrition, Microbiota and Role of Gut-Brain Axis in Subjects with Phenylketonuria (PKU): A Review. Nutrients.

[49] Ahring K., Bélanger-Quintana A., Dokoupil K., Gokmen-Ozel H., Lammardo A.M., MacDonald A., Motzfeldt K., Nowacka M., Robert M., van Rijn M. (2011). Blood phenylalanine control in phenylketonuria: A survey of 10 European centres. Eur. J. Clin. Nutr..

