# Supplementary material for: Natural Protein Intake in Children with Phenylketonuria: Prescription vs. Actual Intakes
Source: Nutrients. 2023 Nov 23;15(23):4903. doi: 10.3390/nu15234903 (PMC10708375; doi:10.3390/nu15234903)
Supplement: Supplementary file 1 [file nutrients-15-04903-s001.zip › nutrients-2712115-supplementary.pdf]

Supplementary Table S1. Natural protein and phenylalanine of all patients during the study – intake and prescribed.

| Subject number (Month) (Day) | Prescribed natural protein intake (g) | Actual total Natural protein intake (g) | Difference between natural protein prescription vs. intake (g) | Prescribed phenylalanine (mg) | Actual total Phenylalanine intake (mg) | Difference between phenylalanine prescription vs. intake (mg) | % increase of Natural protein intake vs prescribed |
|---|---|---|---|---|---|---|---|
| **1** (Month 1) (Day 1) | 5 | 8 | 3 | 250 | 400 | 150 | 38 |
| **1** (Month 1) (Day 2) | 5 | 8 | 3 | 250 | 400 | 150 | 38 |
| **1** (Month 1) (Day 3) | 5 | 10 | 5 | 250 | 500 | 250 | 50 |
| **1** (Month 2) (Day 1) | 5 | 9 | 4 | 250 | 450 | 200 | 44 |
| **1** (Month 2) (Day 2) | 5 | 10 | 5 | 250 | 500 | 250 | 50 |
| **1** (Month 2) (Day 3) | 5 | 11 | 6 | 250 | 550 | 300 | 55 |
| **1** (Month 3) (Day 1) | 5 | 12 | 7 | 250 | 600 | 350 | 58 |
| **1** (Month 3) (Day 2) | 5 | 10 | 5 | 250 | 500 | 250 | 50 |
| **1** (Month 3) (Day 3) | 5 | 11 | 6 | 250 | 550 | 300 | 55 |
| **1** (Month 4) (Day 1) | 5 | 10 | 5 | 250 | 500 | 250 | 50 |
| **1** (Month 4) (Day 2) | 5 | 8 | 3 | 250 | 400 | 150 | 38 |
| **1** (Month 4) (Day 3) | 5 | 11 | 6 | 250 | 550 | 300 | 55 |
| **1** (Month 5) (Day 1) | 5 | 12 | 7 | 250 | 600 | 350 | 58 |
| **1** (Month 5) | 5 | 10 | 5 | 250 | 500 | 250 | 50 |

| | | | | | | | |
|---|---|---|---|---|---|---|---|
| **(Day 2)** | | | | | | | |
| **1**<br>**(Month 5)**<br>**(Day 3)** | 5 | 10 | 5 | 250 | 500 | 250 | 50 |
| **1**<br>**(Month 6)**<br>**(Day 1)** | 5 | 8 | 3 | 250 | 400 | 150 | 38 |
| **1**<br>**(Month 6)**<br>**(Day 2)** | 5 | 9 | 4 | 250 | 450 | 200 | 44 |
| **1**<br>**(Month 6)**<br>**(Day 3)** | 5 | 8 | 3 | 250 | 450 | 200 | 38 |
| 2<br>**(Month 1)**<br>**(Day 1)** | 6,5 | 10 | 3,5 | 325 | 500 | 175 | 35 |
| 2<br>**(Month 1)**<br>**(Day 2)** | 6,5 | 12 | 5,5 | 325 | 600 | 275 | 46 |
| 2<br>**(Month 1)**<br>**(Day 3)** | 6,5 | 9 | 2,5 | 325 | 450 | 125 | 28 |
| 2<br>**(Month 2)**<br>**(Day 1)** | 6,5 | 9 | 2,5 | 325 | 450 | 125 | 28 |
| 2<br>**(Month 2)**<br>**(Day 2)** | 6,5 | 9 | 2,5 | 325 | 450 | 125 | 28 |
| 2<br>**(Month 2)**<br>**(Day 3)** | 6,5 | 12 | 5,5 | 325 | 450 | 125 | 46 |
| 2<br>**(Month 3)**<br>**(Day 1)** | 6,5 | 10 | 3,5 | 325 | 500 | 175 | 35 |
| 2<br>**(Month 3)**<br>**(Day 2)** | 6,5 | 10 | 3,5 | 325 | 500 | 175 | 35 |
| 2<br>**(Month 3)**<br>**(Day 3)** | 6,5 | 9 | 2,5 | 325 | 450 | 125 | 28 |
| 2<br>**(Month 4)**<br>**(Day 1)** | 6,5 | 10 | 3,5 | 325 | 500 | 175 | 35 |
| 2<br>**(Month 4)**<br>**(Day 2)** | 6,5 | 9 | 2,5 | 325 | 450 | 125 | 28 |
| 2<br>**(Month 4)**<br>**(Day 3)** | 6,5 | 10 | 3,5 | 325 | 500 | 175 | 35 |
| 2<br>**(Month 5)**<br>**(Day 1)** | 6,5 | 9 | 2,5 | 325 | 450 | 125 | 28 |

| | | | | | | | |
|---|---|---|---|---|---|---|---|
| 2 **(Month 5) (Day 2)** | 6,5 | 10 | 3,5 | 325 | 500 | 175 | 35 |
| 2 **(Month 5) (Day 3)** | 6,5 | 13 | 6,5 | 325 | 650 | 325 | 50 |
| 2 **(Month 6) (Day 1)** | 6,5 | 9 | 2,5 | 325 | 450 | 125 | 28 |
| 2 **(Month 6) (Day 2)** | 6,5 | 10 | 3,5 | 325 | 500 | 175 | 35 |
| 2 **(Month 6) (Day 3)** | 6,5 | 9 | 2,5 | 325 | 450 | 125 | 28 |
| 3 **(Month 1) (Day 1)** | 27 | 30 | 3 | 1350 | 1500 | 150 | 10 |
| 3 **(Month 1) (Day 2)** | 27 | 30 | 3 | 1350 | 1500 | 150 | 10 |
| 3 **(Month 1) (Day 3)** | 27 | 31 | 4 | 1350 | 1550 | 200 | 13 |
| 3 **(Month 2) (Day 1)** | 27 | 35 | 8 | 1350 | 1750 | 400 | 23 |
| 3 **(Month 2) (Day 2)** | 27 | 32 | 5 | 1350 | 1600 | 250 | 16 |
| 3 **(Month 2) (Day 3)** | 27 | 31 | 4 | 1350 | 1550 | 200 | 13 |
| 3 **(Month 3) (Day 1)** | 27 | 33 | 6 | 1350 | 1650 | 300 | 18 |
| 3 **(Month 3) (Day 2)** | 27 | 33 | 6 | 1350 | 1650 | 300 | 18 |
| 3 **(Month 3) (Day 3)** | 27 | 32 | 5 | 1350 | 1600 | 250 | 16 |
| 3 **(Month 4) (Day 1)** | 27 | 30 | 3 | 1350 | 1500 | 150 | 10 |
| 3 **(Month 4) (Day 2)** | 27 | 34 | 7 | 1350 | 1700 | 350 | 21 |
| 3 **(Month 4) (Day 3)** | 27 | 30 | 3 | 1350 | 1500 | 150 | 10 |
| 3 | 27 | 30 | 3 | 1350 | 1500 | 150 | 10 |

| | | | | | | | |
|---|---|---|---|---|---|---|---|
| **(Month 5)** **(Day 1)** | | | | | | | |
| 3 **(Month 5)** **(Day 2)** | 27 | 30 | 3 | 1350 | 1500 | 150 | 10 |
| 3 **(Month 5)** **(Day 3)** | 27 | 31 | 4 | 1350 | 1550 | 200 | 13 |
| 3 **(Month 6)** **(Day 1)** | 27 | 36 | 9 | 1350 | 1800 | 450 | 25 |
| 3 **(Month 6)** **(Day 2)** | 27 | 37 | 10 | 1350 | 1850 | 500 | 27 |
| 3 **(Month 6)** **(Day 3)** | 27 | 36 | 9 | 1350 | 1800 | 450 | 25 |
| 4 **(Month 1)** **(Day 1)** | 14 | 17 | 3 | 700 | 850 | 150 | 18 |
| 4 **(Month 1)** **(Day 2)** | 14 | 16 | 2 | 700 | 800 | 100 | 13 |
| 4 **(Month 1)** **(Day 3)** | 14 | 23 | 9 | 700 | 1150 | 450 | 39 |
| 4 **(Month 2)** **(Day 1)** | 14 | 17 | 3 | 700 | 850 | 150 | 18 |
| 4 **(Month 2)** **(Day 2)** | 14 | 17 | 3 | 700 | 850 | 150 | 18 |
| 4 **(Month 2)** **(Day 3)** | 14 | 19 | 5 | 700 | 950 | 250 | 26 |
| 4 **(Month 3)** **(Day 1)** | 14 | 17 | 3 | 700 | 850 | 150 | 18 |
| 4 **(Month 3)** **(Day 2)** | 14 | 19 | 5 | 700 | 950 | 250 | 26 |
| 4 **(Month 3)** **(Day 3)** | 14 | 19 | 5 | 700 | 950 | 250 | 26 |
| 4 **(Month 4)** **(Day 1)** | 14 | 16 | 2 | 700 | 800 | 100 | 13 |
| 4 **(Month 4)** **(Day 2)** | 14 | 16 | 2 | 700 | 650 | -50 | 13 |
| 4 **(Month 4)** | 14 | 13 | -1 | 700 | 850 | 150 | -8 |

| | | | | | | | |
|---|---|---|---|---|---|---|---|
| (Day 3) | | | | | | | |
| 4 (Month 5) (Day 1) | 14 | 17 | 3 | 700 | 850 | 150 | 18 |
| 4 (Month 5) (Day 2) | 14 | 17 | 3 | 700 | 900 | 200 | 18 |
| 4 (Month 5) (Day 3) | 14 | 19 | 5 | 700 | 850 | 150 | 26 |
| 4 (Month 6) (Day 1) | 14 | 17 | 3 | 700 | 900 | 200 | 18 |
| 4 (Month 6) (Day 2) | 14 | 19 | 5 | 700 | 850 | 150 | 26 |
| 4 (Month 6) (Day 3) | 14 | 19 | 5 | 700 | 850 | 150 | 26 |
| 5 (Month 1) (Day 1) | 6 | 7 | 1 | 300 | 350 | 50 | 14 |
| 5 (Month 1) (Day 2) | 6 | 10 | 4 | 300 | 500 | 200 | 40 |
| 5 (Month 1) (Day 3) | 6 | 8 | 2 | 300 | 400 | 100 | 25 |
| 5 (Month 2) (Day 1) | 6 | 9 | 3 | 300 | 250 | -50 | 33 |
| 5 (Month 2) (Day 2) | 6 | 5 | -1 | 300 | 450 | 150 | -20 |
| 5 (Month 2) (Day 3) | 6 | 9 | 3 | 300 | 450 | 150 | 33 |
| 5 (Month 3) (Day 1) | 6 | 10 | 4 | 300 | 350 | 50 | 40 |
| 5 (Month 3) (Day 2) | 6 | 9 | 3 | 300 | 450 | 150 | 33 |
| 5 (Month 3) (Day 3) | 6 | 7 | 1 | 300 | 350 | 50 | 14 |
| 5 (Month 4) (Day 1) | 6 | 7 | 1 | 300 | 350 | 50 | 14 |
| 5 (Month 4) (Day 2) | 6 | 9 | 3 | 300 | 450 | 150 | 33 |

| | | | | | | | |
|---|---|---|---|---|---|---|---|
| 5 **(Month 4)** **(Day 3)** | 6 | 7 | 1 | 300 | 350 | 50 | 14 |
| 5 **(Month 5)** **(Day 1)** | 6 | 11 | 5 | 300 | 550 | 250 | 45 |
| 5 **(Month 5)** **(Day 2)** | 6 | 9 | 3 | 300 | 450 | 150 | 33 |
| 5 **(Month 5)** **(Day 3)** | 6 | 7 | 1 | 300 | 450 | 150 | 14 |
| 5 **(Month 6)** **(Day 1)** | 6 | 9 | 3 | 300 | 450 | 150 | 33 |
| 5 **(Month 6)** **(Day 2)** | 6 | 12 | 6 | 300 | 600 | 300 | 50 |
| 5 **(Month 6)** **(Day 3)** | 6 | 10 | 4 | 300 | 500 | 200 | 40 |
| 6 **(Month 1)** **(Day 1)** | 7,5 | 5 | -2,5 | 375 | 250 | -125 | -50 |
| 6 **(Month 1)** **(Day 2)** | 7,5 | 9 | 1,5 | 375 | 450 | 75 | 17 |
| 6 **(Month 1)** **(Day 3)** | 7,5 | 12 | 4,5 | 375 | 600 | 225 | 38 |
| 6 **(Month 2)** **(Day 1)** | 7,5 | 7 | -0,5 | 375 | 350 | -25 | -7 |
| 6 **(Month 2)** **(Day 2)** | 7,5 | 11 | 3,5 | 375 | 550 | 175 | 32 |
| 6 **(Month 2)** **(Day 3)** | 7,5 | 12 | 4,5 | 375 | 600 | 225 | 38 |
| 6 **(Month 3)** **(Day 1)** | 7,5 | 5 | -2,5 | 375 | 250 | -125 | -50 |
| 6 **(Month 3)** **(Day 2)** | 7,5 | 9 | 1,5 | 375 | 450 | 75 | 17 |
| 6 **(Month 3)** **(Day 3)** | 7,5 | 12 | 4,5 | 375 | 600 | 225 | 38 |
| 6 **(Month 4)** **(Day 1)** | 7,5 | 14 | 6,5 | 375 | 700 | 325 | 46 |
| 6 | 7,5 | 11 | 3,5 | 375 | 550 | 175 | 32 |

| | | | | | | | |
|---|---|---|---|---|---|---|---|
| **(Month 4)** **(Day 2)** | | | | | | | |
| 6 **(Month 4)** **(Day 3)** | 7,5 | 14 | 6,5 | 375 | 700 | 325 | 46 |
| 6 **(Month 5)** **(Day 1)** | 7,5 | 7 | -0,5 | 375 | 350 | -25 | -7 |
| 6 **(Month 5)** **(Day 2)** | 7,5 | 11 | 3,5 | 375 | 550 | 175 | 32 |
| 6 **(Month 5)** **(Day 3)** | 7,5 | 12 | 4,5 | 375 | 600 | 225 | 38 |
| 6 **(Month 6)** **(Day 1)** | 7,5 | 9 | 1,5 | 375 | 450 | 75 | 17 |
| 6 **(Month 6)** **(Day 2)** | 7,5 | 9 | 1,5 | 375 | 450 | 75 | 17 |
| 6 **(Month 6)** **(Day 3)** | 7,5 | 12 | 4,5 | 375 | 600 | 225 | 38 |
| 7 **(Month 1)** **(Day 1)** | 4,5 | 9 | 4,5 | 225 | 250 | 25 | 50 |
| 7 **(Month 1)** **(Day 2)** | 4,5 | 5 | 0,5 | 225 | 500 | 275 | 10 |
| 7 **(Month 1)** **(Day 3)** | 4,5 | 10 | 5,5 | 225 | 450 | 225 | 55 |
| 7 **(Month 2)** **(Day 1)** | 4,5 | 9 | 4,5 | 225 | 800 | 575 | 50 |
| 7 **(Month 2)** **(Day 2)** | 4,5 | 16 | 11,5 | 225 | 500 | 275 | 72 |
| 7 **(Month 2)** **(Day 3)** | 4,5 | 10 | 5,5 | 225 | 450 | 225 | 55 |
| 7 **(Month 3)** **(Day 1)** | 4,5 | 9 | 4,5 | 225 | 250 | 25 | 50 |
| 7 **(Month 3)** **(Day 2)** | 4,5 | 5 | 0,5 | 225 | 500 | 275 | 10 |
| 7 **(Month 3)** **(Day 3)** | 4,5 | 10 | 5,5 | 225 | 300 | 75 | 55 |
| 7 **(Month 4)** | 4,5 | 6 | 1,5 | 225 | 200 | -25 | 25 |

| | | | | | | | |
|---|---|---|---|---|---|---|---|
| (Day 1) | | | | | | | |
| 7 (Month 4) (Day 2) | 4,5 | 8 | 3,5 | 225 | 400 | 175 | 44 |
| 7 (Month 4) (Day 3) | 4,5 | 7 | 2,5 | 225 | 350 | 125 | 36 |
| 7 (Month 5) (Day 1) | 4,5 | 9 | 4,5 | 225 | 450 | 225 | 50 |
| 7 (Month 5) (Day 2) | 4,5 | 5 | 0,5 | 225 | 250 | 25 | 10 |
| 7 (Month 5) (Day 3) | 4,5 | 10 | 5,5 | 225 | 500 | 275 | 55 |
| 7 (Month 6) (Day 1) | 4,5 | 9 | 4,5 | 225 | 450 | 225 | 50 |
| 7 (Month 6) (Day 2) | 4,5 | 16 | 11,5 | 225 | 800 | 575 | 72 |
| 7 (Month 6) (Day 3) | 4,5 | 10 | 5,5 | 225 | 500 | 275 | 55 |
| 8 (Month 1) (Day 1) | 3 | 11 | 8 | 150 | 550 | 400 | 73 |
| 8 (Month 1) (Day 2) | 3 | 11 | 8 | 150 | 550 | 400 | 73 |
| 8 (Month 1) (Day 3) | 3 | 10 | 7 | 150 | 500 | 350 | 70 |
| 8 (Month 2) (Day 1) | 3 | 7 | 4 | 150 | 350 | 200 | 57 |
| 8 (Month 2) (Day 2) | 3 | 9 | 6 | 150 | 450 | 300 | 67 |
| 8 (Month 2) (Day 3) | 3 | 4 | 1 | 150 | 200 | 50 | 25 |
| 8 (Month 3) (Day 1) | 3 | 5 | 2 | 150 | 250 | 100 | 40 |
| 8 (Month 3) (Day 2) | 3 | 5 | 2 | 150 | 250 | 100 | 40 |
| 8 (Month 3) (Day 3) | 3 | 7 | 4 | 150 | 350 | 200 | 57 |

| | | | | | | | |
|---|---|---|---|---|---|---|---|
| 8 **(Month 4) (Day 1)** | 3 | 11 | 8 | 150 | 550 | 400 | 73 |
| 8 **(Month 4) (Day 3)** | 3 | 11 | 8 | 150 | 550 | 400 | 73 |
| 8 **(Month 4) (Day 4)** | 3 | 10 | 7 | 150 | 500 | 350 | 70 |
| 8 **(Month 5) (Day 1)** | 3 | 8 | 5 | 150 | 400 | 250 | 63 |
| 8 **(Month 5) (Day 2)** | 3 | 6 | 3 | 150 | 300 | 150 | 50 |
| 8 **(Month 5) (Day 3)** | 3 | 4 | 1 | 150 | 200 | 50 | 25 |
| 8 **(Month 6) (Day 1)** | 3 | 7 | 4 | 150 | 350 | 200 | 57 |
| 8 **(Month 6) (Day 2)** | 3 | 9 | 6 | 150 | 450 | 300 | 67 |
| 8 **(Month 6) (Day 3)** | 3 | 4 | 1 | 150 | 200 | 50 | 25 |
| 9 **(Month 1) (Day 1)** | 5 | 8 | 3 | 250 | 400 | 150 | 38 |
| 9 **(Month 1) (Day 2)** | 5 | 9 | 4 | 250 | 450 | 200 | 44 |
| 9 **(Month 1) (Day 3)** | 5 | 10 | 5 | 250 | 500 | 250 | 50 |
| 9 **(Month 2) (Day 1)** | 5 | 9 | 4 | 250 | 450 | 200 | 44 |
| 9 **(Month 2) (Day 2)** | 5 | 10 | 5 | 250 | 500 | 250 | 50 |
| 9 **(Month 2) (Day 3)** | 5 | 10 | 5 | 250 | 500 | 250 | 50 |
| 9 **(Month 3) (Day 1)** | 5 | 12 | 7 | 250 | 600 | 350 | 58 |
| 9 **(Month 3) (Day 2)** | 5 | 10 | 5 | 250 | 500 | 250 | 50 |
| 9 | 5 | 10 | 5 | 250 | 500 | 250 | 50 |

| | | | | | | | |
|---|---|---|---|---|---|---|---|
| **(Month 3)** **(Day 3)** | | | | | | | |
| 9 **(Month 4)** **(Day 1)** | 5 | 9 | 4 | 250 | 450 | 200 | 44 |
| 9 **(Month 4)** **(Day 2)** | 5 | 8 | 3 | 250 | 400 | 150 | 38 |
| 9 **(Month 4)** **(Day 3)** | 5 | 7 | 2 | 250 | 350 | 100 | 29 |
| 9 **(Month 5)** **(Day 1)** | 5 | 8 | 3 | 250 | 400 | 150 | 38 |
| 9 **(Month 5)** **(Day 2)** | 5 | 6 | 1 | 250 | 300 | 50 | 17 |
| 9 **(Month 5)** **(Day 3)** | 5 | 10 | 5 | 250 | 500 | 250 | 50 |
| 9 **(Month 6)** **(Day 1)** | 5 | 9 | 4 | 250 | 450 | 200 | 44 |
| 9 **(Month 6)** **(Day 2)** | 5 | 8 | 3 | 250 | 400 | 150 | 38 |
| 9 **(Month 6)** **(Day 3)** | 5 | 8 | 3 | 250 | 400 | 150 | 38 |
| 10 **(Month 1)** **(Day 1)** | 4 | 6 | 2 | 200 | 300 | 100 | 33 |
| 10 **(Month 1)** **(Day 2)** | 4 | 7 | 3 | 200 | 350 | 150 | 43 |
| 10 **(Month 1)** **(Day 3)** | 4 | 8 | 4 | 200 | 400 | 200 | 50 |
| 10 **(Month 2)** **(Day 1)** | 4 | 8 | 4 | 200 | 400 | 200 | 50 |
| 10 **(Month 2)** **(Day 2)** | 4 | 7 | 3 | 200 | 350 | 150 | 43 |
| 10 **(Month 2)** **(Day 3)** | 4 | 4 | 0 | 200 | 200 | 0 | 0 |
| 10 **(Month 3)** **(Day 1)** | 4 | 13 | 9 | 200 | 650 | 450 | 69 |
| 10 **(Month 3)** | 4 | 9 | 5 | 200 | 450 | 250 | 56 |

| | | | | | | | |
|---|---|---|---|---|---|---|---|
| **(Day 2)** | | | | | | | |
| 10 **(Month 3) (Day 3)** | 4 | 9 | 5 | 200 | 450 | 250 | 56 |
| 10 **(Month 4) (Day 1)** | 4 | 4 | 0 | 200 | 200 | 0 | 0 |
| 10 **(Month 4) (Day 2)** | 4 | 9 | 5 | 200 | 450 | 250 | 56 |
| 10 **(Month 4) (Day 3)** | 4 | 8 | 4 | 200 | 400 | 200 | 50 |
| 10 **(Month 5) (Day 1)** | 4 | 6 | 2 | 200 | 300 | 100 | 33 |
| 10 **(Month 5) (Day 2)** | 4 | 8 | 4 | 200 | 400 | 200 | 50 |
| 10 **(Month 5) (Day 3)** | 4 | 6 | 2 | 200 | 300 | 100 | 33 |
| 10 **(Month 6) (Day 1)** | 4 | 5 | 1 | 200 | 250 | 50 | 20 |
| 10 **(Month 6) (Day 2)** | 4 | 7 | 3 | 200 | 350 | 150 | 43 |
| 10 **(Month 6) (Day 3)** | 4 | 6 | 2 | 200 | 300 | 100 | 33 |
| 11 **(Month 1) (Day 1)** | 8 | 13 | 5 | 400 | 650 | 250 | 38 |
| 11 **(Month 1) (Day 2)** | 8 | 10 | 2 | 400 | 500 | 100 | 20 |
| 11 **(Month 2) (Day 1)** | 8 | 16 | 8 | 400 | 800 | 400 | 50 |
| 11 **(Month 2) (Day 2)** | 8 | 12 | 4 | 400 | 600 | 200 | 33 |
| 11 **(Month 3) (Day 1)** | 8 | 12 | 4 | 400 | 600 | 200 | 33 |
| 11 **(Month 3) (Day 2)** | 8 | 14 | 6 | 400 | 700 | 300 | 43 |
| 11 **(Month 4) (Day 1)** | 8 | 14 | 6 | 400 | 700 | 300 | 43 |

| | | | | | | | |
|---|---|---|---|---|---|---|---|
| 11 **(Month 4) (Day 2)** | 8 | 11 | 3 | 400 | 550 | 150 | 27 |
| 11 **(Month 5) (Day 1)** | 8 | 12 | 4 | 400 | 600 | 200 | 33 |
| 11 **(Month 5) (Day 2)** | 8 | 11 | 3 | 400 | 550 | 150 | 27 |
| 11 **(Month 1) (Day 1)** | 8 | 7 | -1 | 400 | 350 | -50 | -14 |
| 11 **(Month 6) (Day 2)** | 8 | 8 | 0 | 400 | 400 | 0 | 0 |
| 12 **(Month 1) (Day 1)** | 4 | 10 | 6 | 200 | 500 | 300 | 60 |
| 12 **(Month 1) (Day 2)** | 4 | 9 | 5 | 200 | 450 | 250 | 56 |
| 12 **(Month 1) (Day 3)** | 4 | 13 | 9 | 200 | 650 | 450 | 69 |
| 12 **(Month 2) (Day 1)** | 4 | 5 | 1 | 200 | 250 | 50 | 20 |
| 12 **(Month 2) (Day 2)** | 4 | 7 | 3 | 200 | 350 | 150 | 43 |
| 12 **(Month 2) (Day 3)** | 4 | 8 | 4 | 200 | 400 | 200 | 50 |
| 12 **(Month 3) (Day 1)** | 4 | 5 | 1 | 200 | 250 | 50 | 20 |
| 12 **(Month 3) (Day 2)** | 4 | 9 | 5 | 200 | 450 | 250 | 56 |
| 12 **(Month 3) (Day 3)** | 4 | 9 | 5 | 200 | 450 | 250 | 56 |
| 12 **(Month 4) (Day 1)** | 4 | 9 | 5 | 200 | 450 | 250 | 56 |
| 12 **(Month 4) (Day 2)** | 4 | 5 | 1 | 200 | 250 | 50 | 20 |
| 12 **(Month 4) (Day 3)** | 4 | 5 | 1 | 200 | 250 | 50 | 20 |
| 12 | 4 | 4 | 0 | 200 | 200 | 0 | 0 |

| | | | | | | | |
|---|---|---|---|---|---|---|---|
| **(Month 5)** **(Day 1)** | | | | | | | |
| 12 **(Month 5)** **(Day 2)** | 4 | 8 | 4 | 200 | 400 | 200 | 50 |
| 12 **(Month 5)** **(Day 3)** | 4 | 9 | 5 | 200 | 450 | 250 | 56 |
| 12 **(Month 6)** **(Day 1)** | 4 | 12 | 8 | 200 | 600 | 400 | 67 |
| 12 **(Month 6)** **(Day 2)** | 4 | 9 | 5 | 200 | 450 | 250 | 56 |
| 12 **(Month 6)** **(Day 3)** | 4 | 9 | 5 | 200 | 450 | 250 | 56 |
| 13 **(Month 1)** **(Day 1)** | 7 | 15 | 8 | 350 | 750 | 400 | 53 |
| 13 **(Month 1)** **(Day 2)** | 7 | 10 | 3 | 350 | 500 | 150 | 30 |
| 13 **(Month 2)** **(Day 1)** | 7 | 13 | 6 | 350 | 650 | 300 | 46 |
| 13 **(Month 2)** **(Day 2)** | 7 | 9 | 2 | 350 | 450 | 100 | 22 |
| 13 **(Month 3)** **(Day 1)** | 7 | 9 | 2 | 350 | 450 | 100 | 22 |
| 13 **(Month 3)** **(Day 2)** | 7 | 13 | 6 | 350 | 650 | 300 | 46 |
| 13 **(Month 4)** **(Day 1)** | 7 | 15 | 8 | 350 | 750 | 400 | 53 |
| 13 **(Month 4)** **(Day 2)** | 7 | 10 | 3 | 350 | 500 | 150 | 30 |
| 13 **(Month 5)** **(Day 1)** | 7 | 10 | 3 | 350 | 500 | 150 | 30 |
| 13 **(Month 5)** **(Day 2)** | 7 | 10 | 3 | 350 | 500 | 150 | 30 |
| 13 **(Month 6)** **(Day 1)** | 7 | 13 | 6 | 350 | 650 | 300 | 46 |
| 13 **(Month 6)** | 7 | 9 | 2 | 350 | 450 | 100 | 22 |

| | | | | | | | |
|---|---|---|---|---|---|---|---|
| (Day 2) | | | | | | | |
| 14 **(Month 1)** **(Day 1)** | 6 | 10 | 4 | 300 | 500 | 200 | 40 |
| 14 **(Month 1)** **(Day 2)** | 6 | 9 | 3 | 300 | 450 | 150 | 33 |
| 14 **(Month 1)** **(Day 3)** | 6 | 12 | 6 | 300 | 600 | 300 | 50 |
| 14 **(Month 2)** **(Day 1)** | 6 | 9 | 3 | 300 | 450 | 150 | 33 |
| 14 **(Month 2)** **(Day 2)** | 6 | 9 | 3 | 300 | 450 | 150 | 33 |
| 14 **(Month 2)** **(Day 3)** | 6 | 7 | 1 | 300 | 350 | 50 | 14 |
| 14 **(Month 3)** **(Day 1)** | 6 | 10 | 4 | 300 | 500 | 200 | 40 |
| 14 **(Month 3)** **(Day 2)** | 6 | 10 | 4 | 300 | 500 | 200 | 40 |
| 14 **(Month 3)** **(Day 2)** | 6 | 7 | 1 | 300 | 350 | 50 | 14 |
| 14 **(Month 4)** **(Day 1)** | 6 | 9 | 3 | 300 | 450 | 150 | 33 |
| 14 **(Month 4)** **(Day 2)** | 6 | 8 | 2 | 300 | 400 | 100 | 25 |
| 14 **(Month 4)** **(Day 3)** | 6 | 8 | 2 | 300 | 550 | 250 | 25 |
| 14 **(Month 5)** **(Day 1)** | 6 | 7 | 1 | 300 | 400 | 100 | 14 |
| 14 **(Month 5)** **(Day 2)** | 6 | 9 | 3 | 300 | 350 | 50 | 33 |
| 14 **(Month 5)** **(Day 3)** | 6 | 9 | 3 | 300 | 450 | 150 | 33 |
| 14 **(Month 6)** **(Day 1)** | 6 | 10 | 4 | 300 | 450 | 150 | 40 |
| 14 **(Month 6)** **(Day 2)** | 6 | 12 | 6 | 300 | 500 | 200 | 50 |

| | | | | | | | |
|---|---|---|---|---|---|---|---|
| 14<br>**(Month 6)**<br>**(Day 3)** | 6 | 12 | 6 | 300 | 600 | 300 | 50 |
| 15<br>**(Month 1)**<br>**(Day 1)** | 4 | 5 | 1 | 200 | 250 | 50 | 20 |
| 15<br>**(Month 1)**<br>**(Day 2)** | 4 | 6 | 2 | 200 | 300 | 100 | 33 |
| 15<br>**(Month 1)**<br>**(Day 3)** | 4 | 6 | 2 | 200 | 300 | 100 | 33 |
| 15<br>**(Month 2)**<br>**(Day 1)** | 4 | 5 | 1 | 200 | 250 | 50 | 20 |
| 15<br>**(Month 2)**<br>**(Day 2)** | 4 | 9 | 5 | 200 | 450 | 250 | 56 |
| 15<br>**(Month 2)**<br>**(Day 3)** | 4 | 6 | 2 | 200 | 300 | 100 | 33 |
| 15<br>**(Month 1)**<br>**(Day 1)** | 4 | 9 | 5 | 200 | 450 | 250 | 56 |
| 15<br>**(Month 2)**<br>**(Day 2)** | 4 | 9 | 5 | 200 | 450 | 250 | 56 |
| 15<br>**(Month 3)**<br>**(Day 3)** | 4 | 10 | 6 | 200 | 500 | 300 | 60 |
| 15<br>**(Month 4)**<br>**(Day 1)** | 4 | 10 | 6 | 200 | 500 | 300 | 60 |
| 15<br>**(Month 4)**<br>**(Day 2)** | 4 | 5 | 1 | 200 | 250 | 50 | 20 |
| 15<br>**(Month 4)**<br>**(Day 3)** | 4 | 6 | 2 | 200 | 300 | 100 | 33 |
| 15<br>**(Month 5)**<br>**(Day 1)** | 4 | 5 | 1 | 200 | 250 | 50 | 20 |
| 15<br>**(Month 5)**<br>**(Day 2)** | 4 | 6 | 2 | 200 | 300 | 100 | 33 |
| 15<br>**(Month 5)**<br>**(Day 3)** | 4 | 7 | 3 | 200 | 350 | 150 | 43 |
| 15<br>**(Month 6)**<br>**(Day 1)** | 4 | 5 | 1 | 200 | 250 | 50 | 20 |
| 15 | 4 | 6 | 2 | 200 | 300 | 100 | 33 |

| | | | | | | | |
|---|---|---|---|---|---|---|---|
| (Month 6) (Day 2) | | | | | | | |
| 15 **(Month 6) (Day 3)** | 4 | 7 | 3 | 200 | 350 | 150 | 43 |
| 16 **(Month 1) (Day 1)** | 6 | 12 | 6 | 300 | 600 | 300 | 50 |
| 16 **(Month 1) (Day 2)** | 6 | 11 | 5 | 300 | 550 | 250 | 45 |
| 16 **(Month 1) (Day 3)** | 6 | 15 | 9 | 300 | 750 | 450 | 60 |
| 16 **(Month 2) (Day 1)** | 6 | 13 | 7 | 300 | 650 | 350 | 54 |
| 16 **(Month 2) (Day 2)** | 6 | 13 | 7 | 300 | 650 | 350 | 54 |
| 16 **(Month 2) (Day 3)** | 6 | 10 | 4 | 300 | 500 | 200 | 40 |
| 16 **(Month 3) (Day 1)** | 6 | 13 | 7 | 300 | 650 | 350 | 54 |
| 16 **(Month 3) (Day 2)** | 6 | 12 | 6 | 300 | 600 | 300 | 50 |
| 16 **(Month 3) (Day 3)** | 6 | 14 | 8 | 300 | 700 | 400 | 57 |
| 16 **(Month 4) (Day 1)** | 6 | 11 | 5 | 300 | 550 | 250 | 45 |
| 16 **(Month 4) (Day 2)** | 6 | 12 | 6 | 300 | 600 | 300 | 50 |
| 16 **(Month 4) (Day 3)** | 6 | 12 | 6 | 300 | 600 | 300 | 50 |
| 16 **(Month 5) (Day 1)** | 6 | 14 | 8 | 300 | 700 | 400 | 57 |
| 16 **(Month 5) (Day 2)** | 6 | 14 | 8 | 300 | 700 | 400 | 57 |
| 16 **(Month 5) (Day 3)** | 6 | 17 | 11 | 300 | 850 | 550 | 65 |
| 16 **(Month 6)** | 6 | 12 | 6 | 300 | 600 | 300 | 50 |

| | | | | | | | |
|---|---|---|---|---|---|---|---|
| **(Day 1)** | | | | | | | |
| 16 **(Month 6** **(Day 2)** | 6 | 15 | 9 | 300 | 550 | 250 | 60 |
| 16 **(Month 6)** **(Day 3)** | 6 | 15 | 9 | 300 | 750 | 450 | 60 |
